# Genomics analysis of *Drosophila sechellia* response to *Morinda citrifolia* fruit diet

**DOI:** 10.1093/g3journal/jkac153

**Published:** 2022-06-23

**Authors:** Zachary Drum, Stephen Lanno, Sara M Gregory, Serena Shimshak, Will Barr, Austin Gatesman, Mark Schadt, Jack Sanford, Aaron Arkin, Brynn Assignon, Sofia Colorado, Carol Dalgarno, Trevor Devanny, Tara Ghandour, Rose Griffin, Mia Hogan, Erica Horowitz, Emily McGhie, Jake Multer, Hannah O'Halloran, Kofi Ofori-Darko, Dmitry Pokushalov, Nick Richards, Kathleen Sagarin, Nicholas Taylor, Acadia Thielking, Phie Towle, Joseph Coolon

**Affiliations:** Department of Biology, Wesleyan University, Middletown, CT 06457, USA; Department of Biology, Wesleyan University, Middletown, CT 06457, USA; Department of Biology, Wesleyan University, Middletown, CT 06457, USA; Department of Biology, Wesleyan University, Middletown, CT 06457, USA; Department of Biology, Wesleyan University, Middletown, CT 06457, USA; Department of Biology, Wesleyan University, Middletown, CT 06457, USA; Department of Biology, Wesleyan University, Middletown, CT 06457, USA; Department of Biology, Wesleyan University, Middletown, CT 06457, USA; Department of Biology, Wesleyan University, Middletown, CT 06457, USA; Department of Biology, Wesleyan University, Middletown, CT 06457, USA; Department of Biology, Wesleyan University, Middletown, CT 06457, USA; Department of Biology, Wesleyan University, Middletown, CT 06457, USA; Department of Biology, Wesleyan University, Middletown, CT 06457, USA; Department of Biology, Wesleyan University, Middletown, CT 06457, USA; Department of Biology, Wesleyan University, Middletown, CT 06457, USA; Department of Biology, Wesleyan University, Middletown, CT 06457, USA; Department of Biology, Wesleyan University, Middletown, CT 06457, USA; Department of Biology, Wesleyan University, Middletown, CT 06457, USA; Department of Biology, Wesleyan University, Middletown, CT 06457, USA; Department of Biology, Wesleyan University, Middletown, CT 06457, USA; Department of Biology, Wesleyan University, Middletown, CT 06457, USA; Department of Biology, Wesleyan University, Middletown, CT 06457, USA; Department of Biology, Wesleyan University, Middletown, CT 06457, USA; Department of Biology, Wesleyan University, Middletown, CT 06457, USA; Department of Biology, Wesleyan University, Middletown, CT 06457, USA; Department of Biology, Wesleyan University, Middletown, CT 06457, USA; Department of Biology, Wesleyan University, Middletown, CT 06457, USA; Department of Biology, Wesleyan University, Middletown, CT 06457, USA

**Keywords:** toxin resistance, *Morinda citrifolia*, noni, host specialization, RNA-seq

## Abstract

*Drosophila sechellia* is an island endemic host specialist that has evolved to consume the toxic fruit of *Morinda citrifolia*, also known as noni fruit. Recent studies by our group and others have examined genome-wide gene expression responses of fruit flies to individual highly abundant compounds found in noni responsible for the fruit’s unique chemistry and toxicity. In order to relate these reductionist experiments to the gene expression responses to feeding on noni fruit itself, we fed rotten noni fruit to adult female *D. sechellia* and performed RNA-sequencing. Combining the reductionist and more wholistic approaches, we have identified candidate genes that may contribute to each individual compound and those that play a more general role in response to the fruit as a whole. Using the compound specific and general responses, we used transcription factor prediction analyses to identify the regulatory networks and specific regulators involved in the responses to each compound and the fruit itself. The identified genes and regulators represent the possible genetic mechanisms and biochemical pathways that contribute to toxin resistance and noni specialization in *D. sechellia.*

## Introduction

Insects have intimate relationships with plants, ranging from pollination to parasitism, and mimicry to mutualism. One of the most common of these interactions is insect–host plant specialization. A well-studied example of this is the Seychelles Islands endemic fruit fly specialist *Drosophila sechellia* that feeds almost exclusively on the ripe fruit of the *Morinda citrifolia* or noni plant (Tsacas and Bachli 1981; Louis and David 1986; Matute and Ayroles 2014). *Drosophila*  *sechellia* is considered to be a banner species for specialization because its closest relatives are not specialists, and because it relies heavily on *M. citrifolia* at all of its life stages (Louis and David 1986; R’Kha *et al.* 1991, 1997; Lavista-Llanos *et al.* 2014). Additionally, aside from the ease of cultivating fruit flies in a lab, the ability of *D. sechellia* to hybridize with close relatives facilitated early genetic studies (R’Kha *et al.* 1991). Much interest in *D. sechellia* arises from the observation that ripe *M. citrifolia* fruit is highly toxic to other species of fruit flies yet *D. sechellia* is resistant to this toxicity (R’Kha *et al.* 1991; Andrade Lopez *et al.* 2017).

The main toxins of *M. citrifolia* fruit are volatile fatty acids, to which *D. sechellia* has evolved both high resistance and preference (Legal *et al.* 1992, 1994; Farine *et al.* 1996; Dekker *et al.* 2006; Matute and Ayroles, 2014). A number of studies have centered around the mechanisms of this toxin resistance, most with a focus on the fatty acid volatile octanoic acid (OA; R’Kha *et al.* 1991; Jones 1998; Lavista-Llanos *et al.* 2014; Lanno *et al.* 2017, 2019; Peyser *et al.* 2017; Lanno and Coolon 2019). The other primary fatty acid volatile found in noni fruit, hexanoic acid (HA), is also toxic (Farine *et al.* 1996; Peyser *et al.* 2017; Lanno and Coolon 2019) but less so than OA and is responsible for attracting *D. sechellia* to its host (Amlou, Moreteau, and David 1998). In that vein, recent work has shown that *D. sechellia* prefers the fruit of *M. citrifolia*, it has adapted to it nutritionally and relies on it for normal reproduction (Lavista-Llanos *et al.* 2014; Watanabe *et al.* 2019).

*Drosophila*  *sechellia* grows and reproduces better on *M. citrifolia* than other food sources in part because of adaptation to a lower carbohydrate to protein ratio (Watanabe *et al.* 2019), but also because of reliance on *M. citrifolia* for l-DOPA, a dopamine precursor (Lavista-Llanos *et al.* 2014). The results of Lavista-Llanos *et al.* (2014) explain the observation that maternal environment is more important for larval success in *D. sechellia* than genotype—it is the reliance on an external source of l-DOPA. Surprisingly, they also showed that dopamine confers toxin resistance in other Drosophilids, a result corroborated by Lanno *et al.* (2019). Additionally, the toxic environment in *M. citrifolia* fruit increases egg production in *D. sechellia* but decreases egg production in other *Drosophilae* (R’Kha *et al.* 1991). A gene expression study using microarrays found that *D. sechellia* fed *M. citrifolia* have increased expression of genes associated with egg production and fatty acid metabolism (Dworkin and Jones 2009). Additionally, *D. sechellia* have low levels of 3,4-dihydroxyphenylalanine (l-DOPA), a dopamine precursor, relative to other *Drosophila* species, likely due a mutation in the *Catsup* gene, which regulates the synthesis of l-DOPA from tyrosine. *Morinda*  *citrifolia* contains high levels of l-DOPA. When the l-DOPA in *M. citrifolia* is removed, *D. sechellia* produce fewer eggs. Thus, *D. sechellia* supplement their own low levels of l-DOPA with the l-DOPA found in *M. citrifolia* to increase egg production. In addition, this process allows the eggs to survive in the fruit’s toxic environment (Lavista-Llanos *et al.* 2014). Furthermore, *Drosophila*  *melanogaster* and *Drosophila*  *simulans* adult flies fed l-DOPA have increased resistance to OA (Lavista-Llanos *et al.* 2014; Lanno *et al.* 2019).

Many prior studies in other insects have focused on identifying genes important for insect specialization on specific host plants by using a genome-wide transcriptomic approach. This approach has been applied to investigations of insects in response to specific toxins found in their preferred host plant (Wang *et al.* 2015; Lanno *et al.* 2017, 2019; Li *et al.* 2019; Drum *et al.* 2022) while others investigated gene expression responses to the different host plant as a whole (Govind *et al.* 2010; Bansal *et al.* 2014; Hoang *et al.* 2015; Crava *et al.* 2016; De Panis *et al.* 2016; Birnbaum *et al.* 2017; Schweizer *et al.* 2017). Feeding on different food sources therefore differ in many ways (e.g. presence and concentration of many chemicals) but represent a more ecologically relevant condition. In this study, we compared transcriptomes of *D. sechellia* fed on a standard diet to a diet supplemented with rotten *M. citrifolia* fruit and compare to our prior work investigating gene expression responses to single chemicals found in *M. citrifolia* (Lanno *et al.* 2017, 2019; Drum *et al.* 2022). The rotten *M. citrifolia* represents a condition in which l-DOPA is preserved and toxicity to other Drosophilids due to OA and HA volatiles is not because microbial action reduces the volatile fatty acids in the fruit (David *et al.* 1989; Lavista-Llanos *et al.* 2014). We also analyze significantly differentially expressed genes (DEGs) from each treatment using software that examines shared regulatory motifs among DEGs to make predictions about which transcription factors (TFs) may be regulating the expression of DEGs in response to these different treatments. Therefore, we can make inferences about the role of each compound in altering gene expression in *D. sechellia* and compare the transcriptomic responses induced by each compound as well as identify regulatory networks that might be common to all chemical substances.

## Methods

### Fly strains and culture

*Drosophila sechellia* (14021-0428.25) flies were reared at low density on standard cornmeal medium under a 16:8 light:dark cycle maintained at 20°C.

### RNA-sequencing

Adult female 0- to 3-day-old *D. sechellia* 14021-0428.25 flies were fed control food (Carolina Biological Supply) or control food mixed with 1 g rotten *M. citrifolia* fruit pulp for 24 h. The *Morinda* fruit used in this experiment was harvested when fully ripe (white/gray in color and soft) from plants grown on site. The fruit was then aged in 1 L plastic containers with holes in the lid to allow air movement for 7 days at 25°C and 70% relative humidity. Seeds were removed and pulp homogenized before incorporation in food media. After treatment, flies were snap frozen in liquid nitrogen and stored at −80°C until RNA extraction. Three replicates were analyzed per treatment, with 10 flies per replicate, generating 3 control and 3 noni fed samples. RNA was extracted using the Promega SV total RNA extraction system with modified protocol (Promega; Coolon *et al.* 2013). RNA quality was determined using gel electrophoresis (Thermo Fisher Scientific, USA) and NanoDrop spectrophotometer (Thermo Fisher ScientificUSA). RNA was sent to the University of Michigan Sequencing Core Facility where mRNA selection was performed from total RNA using poly(A) selection. cDNA libraries were then sequenced using the Illumina Hiseq 4000 platform.

### BIOL310 genomics analysis

The genomics analysis of RNA-seq data presented in this manuscript was performed by 2 high school, 20 undergraduate, and 3 graduate students as part of a semester-long course at Wesleyan University called Genomics Analysis (BIOL310). This is the fourth such manuscript (see Lanno *et al.* 2017, 2019; Drum *et al.* 2022) generated from this Course-Based Undergraduate Research Experience (CURE) where the aim is to provide early-stage undergraduate students an opportunity for hands-on research experience with active participation in the process of scientific discovery. Students in the course learn through engaging with newly generated genomics data and use cutting-edge genomics analysis and bioinformatics tools engaging in a discovery-based independent study. Every student in the course contributed to every aspect of the analysis including quality control, bioinformatics, statistical analyses, write-up, and interpretation of the findings, providing their own unique perspective of the results and the text written by each and every student was combined into this manuscript with little modification.

**Fig. 1.**
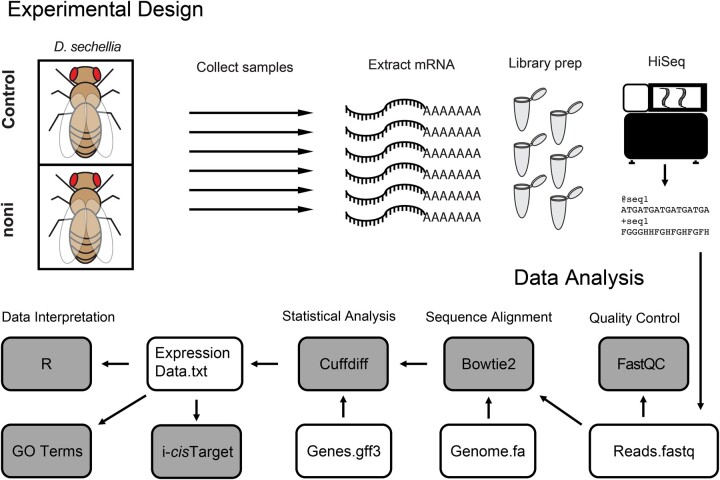
Experimental design and bioinformatics pipeline. Female *D. sechellia* were exposed to either control food or food supplemented with rotten noni fruit. RNA was extracted, underwent polyA selection, library preparation, and sequencing. Raw-sequencing reads were checked for quality using FastQC, and then aligned to the *D. sechellia* reference genome-with Bowtie2. Differential expression testing was performed using Cuffdiff, and expression data were analyzed using R, Gene ontology, and i*cis*-Target.

After sequencing output files were obtained from the University of Michigan Sequencing Core (Table 1), .fastq files containing raw sequencing reads were uploaded to the Galaxy platform (Afgan *et al.* 2016) and an RNA-seq pipeline analysis was performed (Fig. 1) as previously described (Lanno *et al.* 2017, 2019; Drum *et al.* 2022). Briefly, reads were assessed for quality using FASTQC (Andrews 2010) and any overrepresented sequences were analyzed using NCBI Blast (Altschul *et al.* 1990). Bowtie2 was used for mapping reads to the appropriate reference genome for each species with default parameters (Langmead and Salzberg 2012), with the most recent genomes for each species available at the time of analysis acquired from Ensembl (www.ensembl.org, Yates *et al.* 2020; *D. sechellia*: Drosophila_sechellia.dsec_caf1.dna.toplevel.fa). The Bowtie2 output files were analyzed using Cuffdiff (Trapnell *et al.* 2013), for gene expression quantification and differential gene expression analysis using the aforementioned genome file along with the most recent annotated .gff3 file for each genome available at the time of analysis acquired from Ensembl (*D. sechellia*: Drosophila_sechellia.dsec_caf1.42.gff3). In Cuffdiff, geometric normalization and library size correction were performed, along with bias correction using the reference genome, giving an output of DEGs following false discovery rate multiple testing correction (Benjamini and Hochberg 1995, *q* < 0.05). Data were visualized using R. The list of DEGs was uploaded to geneontology.org for Gene Ontology (GO) term enrichment analysis (www.geneontology.org; Ashburner *et al.* 2000; Mi *et al.* 2019; Carbon *et al.* 2021). *Drosophila melanogaster* orthologs for each *D. sechellia* gene were downloaded using FlyBase (Thurmond *et al.* 2019). Data processing and visualization were performed in R (R Core Development Team 2020). The *D. melanogaster* orthologs for each DEGs after feeding on noni food were analyzed through the i-*cis*Target analysis software (https://med.kuleuven.be/lcb/i-cisTarget) to identify putative *cis*-regulatory sequences shared among DEGs (Herrmann *et al.* 2012; Imrichová *et al.* 2015). The top 10 all non-TATA unique sequence elements representing predicted TF binding sites and their downstream targets were then visualized with Cytoscape (https://cytoscape.org/; Shannon *et al.* 2003). DEGs following *D. sechellia* exposure to OA, HA, or l-DOPA were downloaded from the literature (Lanno *et al.* 2017, 2019; Drum *et al.* 2022). Gene overlap testing was performed using the “GeneOverlap” package in R (Shen 2021) with *D. melanogaster* orthologs.

**Table 1.** Samples, mapped reads, and read lengths for each sequencing library.

Sample	ID	No. of reads	No. of mapped reads	% Mapped	Read length (nt)
Control 1	76332	19,222,060	18,496,450	96.23	65
Control 2	76333	20,704,811	19,440,620	93.89	65
Control 3	76334	17,696,868	17,123,579	96.76	65
Noni 1	76350	18,937,596	17,939,488	94.73	65
Noni 2	76351	16,870,429	15,801,506	93.66	65
Noni 3	76352	14,595,340	14,181,949	97.17	65

**Table 2.** DEGs in *D. sechellia* in response to noni treatment.

No. of DEGs	No. of upregulated	No. of downregulated	No. of *D. melanogaster* orthologs
503	179	324	426

## Results

### Identifying genes that are regulated in response to noni fruit diet

**Fig. 2.**
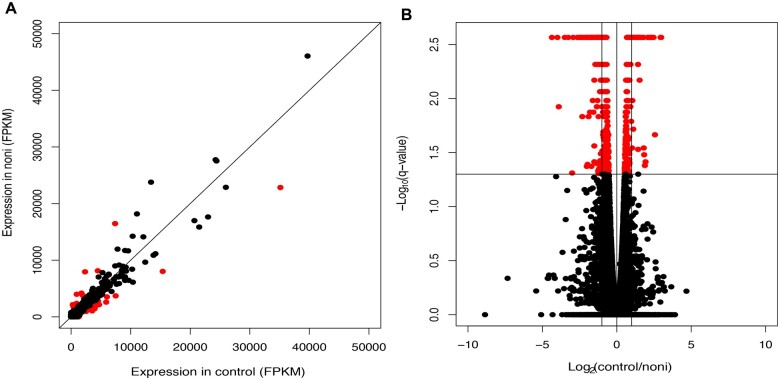
Noni treatment alters genome-wide gene expression in adult *D. sechellia*. a) After RNA-sequencing of *D. sechellia* females fed noni fruit, expression of genes (FPKM) in control are shown on the *X*-axis, with expression of each gene on the *Y*-axis. Genes that are significantly differentially expressed in noni treatment are shown in red. b) Volcano plot of DEGs are shown, with Log_2_(control/noni) on the *X*-axis, and -Log_10_(q-value) on the *Y*-axis. Significantly DEGs are shown in red.

To identify the genes expressed differently when adult *D. sechellia* flies are fed a diet of control food (instant *Drosophila* media) compared to flies fed control food supplemented with rotten noni fruit, we used RNA-seq. Statistical analyses of genome-wide gene expression in these 2 diets identified 503 significantly DEGs (Fig. 2). Of these DEGs, 179 were upregulated in response to noni fruit and 324 DEGs were downregulated (Table 2). Of these 503 DEGs, 421 had annotated *D. melanogaster* orthologs. Of the 82 DEGs without annotated *D. melanogaster* orthologs, 31 DEGs were 5.8S rRNA genes, all of which were downregulated (Table 1). Five of the DEGs had uncertain orthologs based on the presence of paralogs in one or more species were removed for further analysis. For the remainder of the analysis, only genes with *D. melanogaster* orthologs were used so annotation for the identified genes could be utilized.

**Fig. 3.**
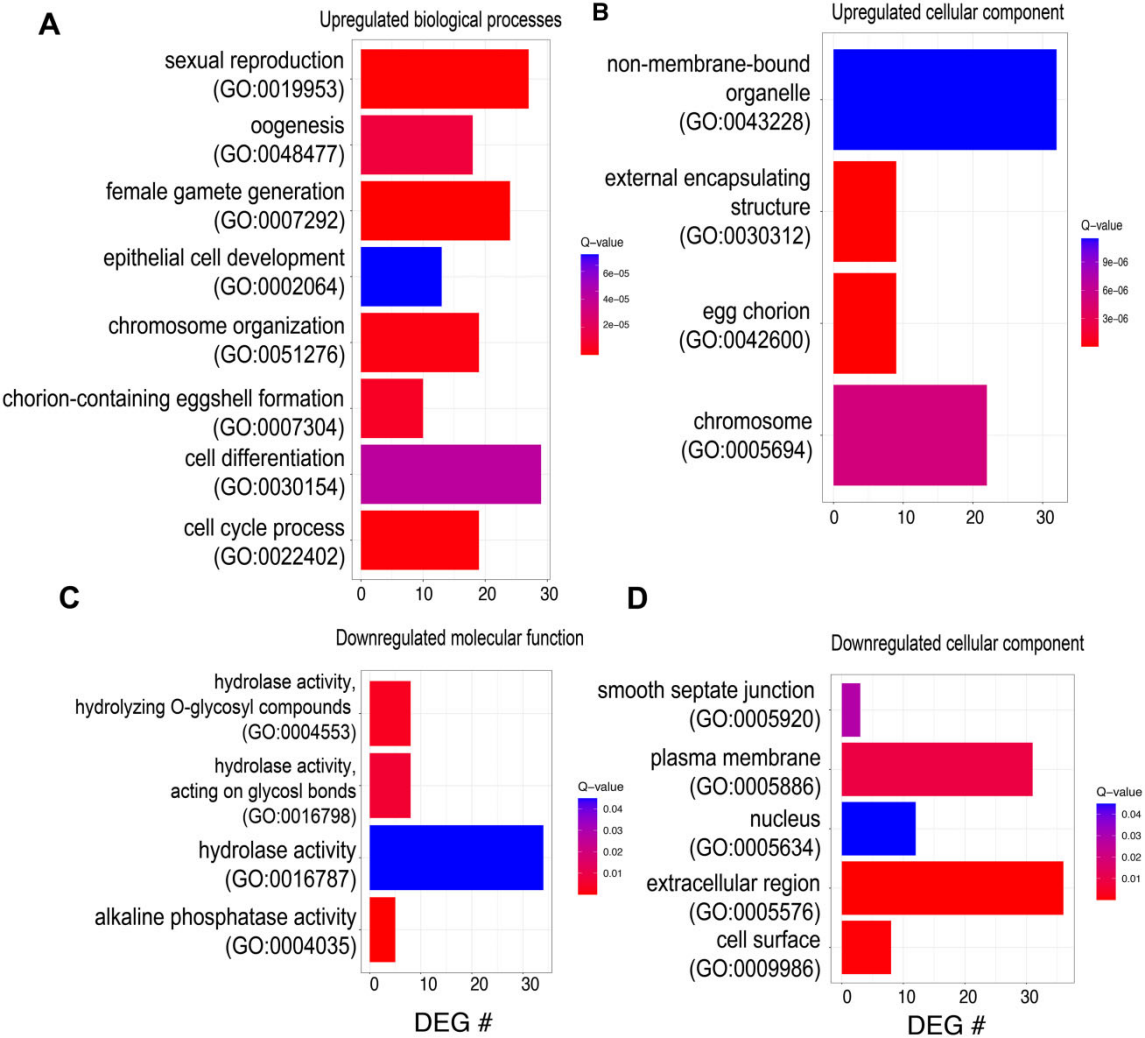
GO analysis of DEGs in response to noni fruit. See Supplementary Tables 7 and 8 for complete lists. a and b) Upregulated DEGs analyzed for enriched GO processes. a) Upregulated DEGs are enriched for several cellular components, including nonmembrane-bound organelle, external encapsulating structure, egg chorion, and chromosome. b) Upregulated DEGs are enriched for several biological processes, including sexual reproductive processes, eggshell formation, and cell cycle processes. c and d) Downregulated DEGs analyzed for enriched GO processes. c) Downregulated DEGs and enriched for several cellular components, including smooth septate junction, plasma membrane, nucleus, extracellular region, and cell surface. d) Downregulated DEGs are enriched for several molecular functions, including hydrolase activity and alkaline phosphatase activity.

In order to identify possible functional enrichment among the genes responsive to noni fruit diet, GO term analysis was performed on up- and downregulated gene sets separately (Fig. 3). The most significantly enriched biological process GO terms from the upregulated DEGs included female gamete generation (GO:0007292, *P* = 1.32e-08), sexual reproduction (GO:0019953, *P* = 3.06e-07), cell cycle process (GO:0022402, *P* = 8.84-07), chromosome organization (GO: 0051276, *P* = 2.20e-06), chorion-containing eggshell formation (GO:0007304, *P* = 6.26e-06), oogenesis (GO:0048477, *P* = 1.22e-05), cell differentiation (GO:0030154, *P* = 4.27e-05), and epithelial cell development (GO:0002064, *P* = 7.22e-05, Fig. 3a) consistent with prior genomics and functional studies in *D. sechellia* that showed increased egg production in response to feeding on noni (R’Kha *et al.* 1991, 1997; Lavista-Llanos *et al.* 2014; Lanno and Coolon 2019). The most significantly enriched cellular component GO terms from upregulated DEGs included egg chorion (GO:0042600, *P* = 3.58e-08), external encapsulating structure (GO:0030312, *P* = 4.43e-08), chromosome (GO:0005694, *P* = 5.04e-06), and nonmembrane-bounded organelle (GO:0043228, *P* = 1.15e-05, Fig. 3b). No significant enrichment of molecular function GO terms was found for upregulated DEGs. The most significantly enriched molecular function GO terms from downregulated genes were alkaline phosphatase activity (GO:0004035, *P* = 2.53e-03), hydrolase activity, hydrolyzing O-glycosyl compounds (GO:0004553, *P* = 5.53e-03), hydrolase activity, acting on glycosyl bonds (GO:0016798, *P* = 8.43e-03), and hydrolase activity (GO:0016787, *P* = 4.66e-02, Fig. 3c). The most significantly enriched cellular component GO terms from downregulated DEGs were extracellular region (GO:0005576, *P* = 4.31e-06), cell surface (GO:0009986, *P* = 4.57e-04), plasma membrane (GO:0005920, *P* = 8.77e-03), smooth septate junction (GO:0005920, *P* = 2.83e-02), and nucleus (GO:0005634, *P* = 4.49e-02, Fig. 3d, Supplementary Tables 7 and 8). No significant enrichment of biological processes GO terms was found for downregulated DEGs.

### Investigating the regulatory network(s) of DEGs responding to noni fruit diet

Identified DEGs were analyzed using i-*cis*Target to determine which TFs may be involved in regulating gene expression upon feeding on rotten noni. Predicted TFs from DEGs that responded to noni treatment were *Adf1*, *GATAd*, *GATAe*, *grn*, *ham*, *pnr*, *sd*, *single-minded* (*sim*), *srp*, and *zld* (Supplementary Fig. 2). This analysis predicted all 5 GATA family TFs as regulators of DEG expression (*GATAd*, *GATAe*, *grn*, *pnr*, *srp*). GATA factors are important in dietary restriction (Dobson *et al.* 2018) and gut stem cell maintenance (Okumura *et al.* 2005), and a possible role in egg formation in insects (Liu *et al.* 2019) making them excellent candidates for roles in evolved responses to altered diet and downstream effects of diet on egg production. Of the predicted TFs responding to noni treatment, only *sim*, a transcriptional repressor involved in nervous system development was significantly upregulated in *D. sechellia* (Estes *et al.* 2001; Supplementary Fig. 1). Of the 31 DEGs predicted to be regulated by *sim* in noni treatment, 23 are downregulated (Supplementary Fig. 2).

### Comparing DEGs responsive to noni fruit, OA, HA, and l-DOPA

**Fig. 4.**
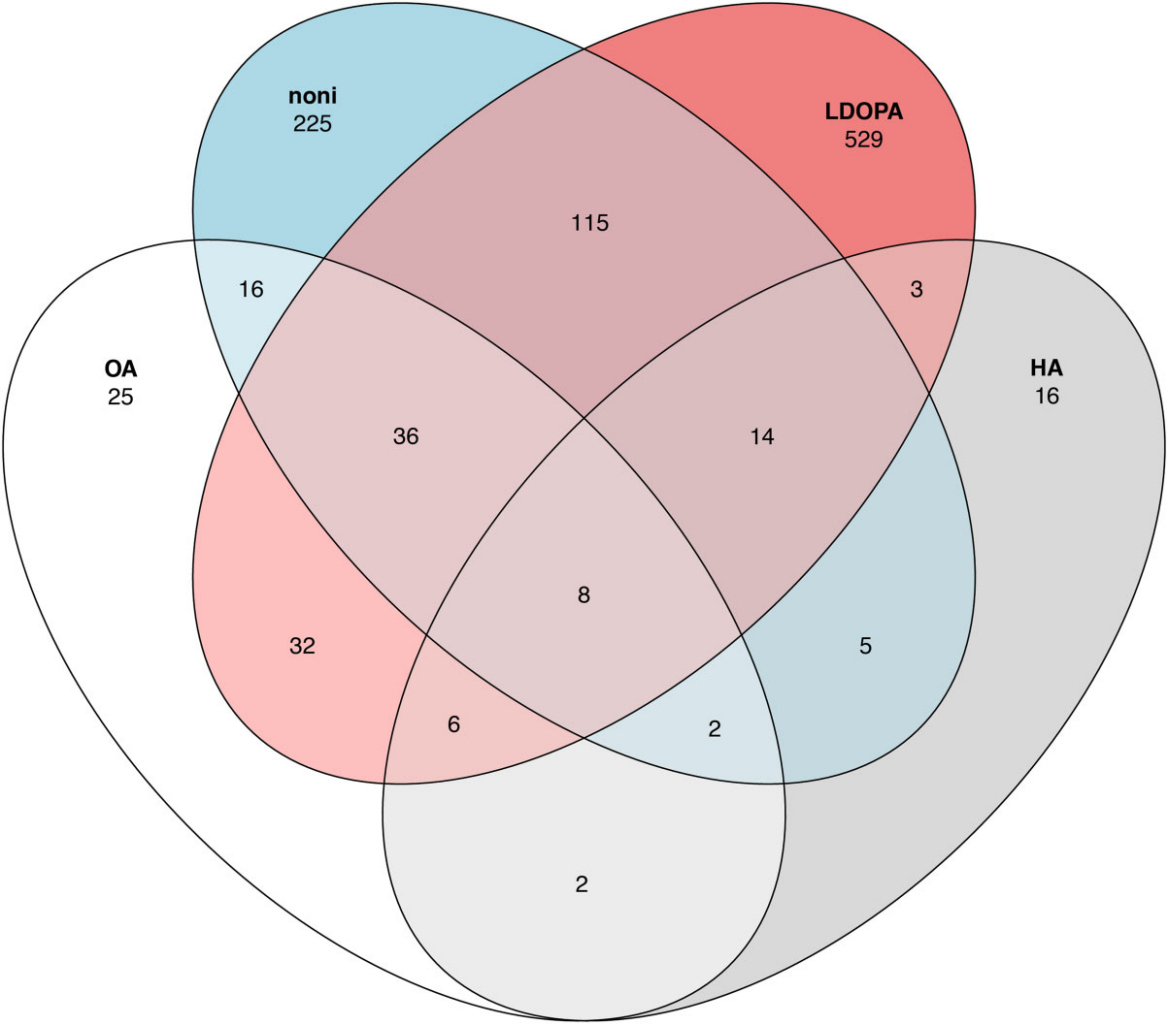
Overlap of significantly DEGs in response to components of noni fruit. DEGs changing expression in response to OA (white), HA (gray), noni (blue), and l-DOPA (red). Overlaps of shared DEGs are shown, as are DEGs specific to each treatment.

The unique niche that *D. sechellia* utilizes by specializing to feed almost solely on noni fruit includes multiple highly abundant plant chemicals including OA, HA, and l-DOPA (Legal *et al.* 1994; Farine *et al.* 1996; Andrade Lopez *et al.* 2017). Genome-wide gene expression investigations of responses to each of these individual chemicals were published previously (Lanno *et al.* 2017, 2019; Drum *et al.* 2022). Comparing the separate transcriptional responses of *D. sechellia* to the individual chemicals with *D. sechellia* fed noni fruit may help elucidate how they evolved to specialize on this toxic fruit. Previous studies investigated the transcriptional response of *D. sechellia* to OA (Lanno *et al.* 2017), HA (Drum *et al.* 2022), and 3,4-dihydroxyphenylalanine (l-DOPA, Lanno *et al.* 2019). DEGs that do not have annotated *D. melanogaster* orthologs in FlyBase were found to be many members of different RNA classes. Upon OA, l-DOPA, and noni treatment, several 5.8SrRNAs, snoRNAs, and 18SrRNAs were all downregulated. In contrast, upon HA treatment several 5.8SrRNAs were upregulated (Supplementary Table 3). Analyzing DEGs in each treatment with annotated *D. melanogaster* orthologs yields 8 DEGs that were significantly differentially expressed in all 4 treatments and all 8 genes were downregulated (Fig. 4). The antimicrobial peptides *Defensin*, *GNBP-like3*, *edin* were significantly downregulated in all 4 treatments, as was the TF *Neu2*, as well as other genes: *Sry-alpha*, *CG14915*, *CG15876*, and *CG6885*. Gene expression responses in *D. sechellia* exposed to rotten noni or l-DOPA treatment yielded 173 genes in common. Of these 173 genes, 149 genes were significantly differentially regulated in the same direction in both treatments. The TF *sim* was upregulated in both noni and l-DOPA treatments. Interestingly, only 25 of 127 genes differentially expressed in OA treatment (Lanno *et al.* 2017) were specific to only OA treatment and not to other compounds from noni fruit. The 2 medium chain fatty acids OA and HA shared only 2 DEGs [*E(spl)mgamma-HLH and AttA*] between them that were specific to only fatty acid treatment and not l-DOPA or rotten noni (Supplementary Tables 1–4).

Overlap significance testing was performed to compare the number of significant DEGs found between each treatment with the “GeneOverlap” package in R (Shen 2021). *Drosophila sechellia* had 17,275 annotated genes in its genome and 13,095 genes with annotated *D. melanogaster* orthologs, so 17,275 was used for the genome size measurement. l-DOPA treatment resulted in 743 DEGs, noni treatment yielded 421 DEGs, OA caused 127 DEGs, and HA treatment resulted in 56 DEGs (Fig. 4). l-DOPA and noni treatments had 173 overlapping DEGs between these treatments (Fisher’s exact test, *P* = 1.9e-127), noni and OA treatments had 62 overlapping DEGs between these treatments (Fisher’s exact test, *P* = 2.9e-66), and noni and HA treatments had 29 overlapping DEGs between these treatments (Fisher’s exact test, *P* = 2.6e-32). We compared between previously analyzed treatments: OA and HA, which had 18 overlapping DEGs (Fisher’s exact test, *P* = 1.9e-25), OA and l-DOPA treatments had 82 overlapping DEGs (Fisher’s exact test, *P* = 9.7e-81), and HA and l-DOPA treatments had 31 overlapping DEGs (Fisher’s exact test, *P* = 4.8e-28). The overlap for every pairwise comparison of DEGs between all 4 treatments was significant suggesting that there may be common regulatory changes that evolved in *D. sechellia* controlling similar gene expression responses to different aspects of their host food species.

### Comparing predicted TFs among treatments

**Fig. 5.**
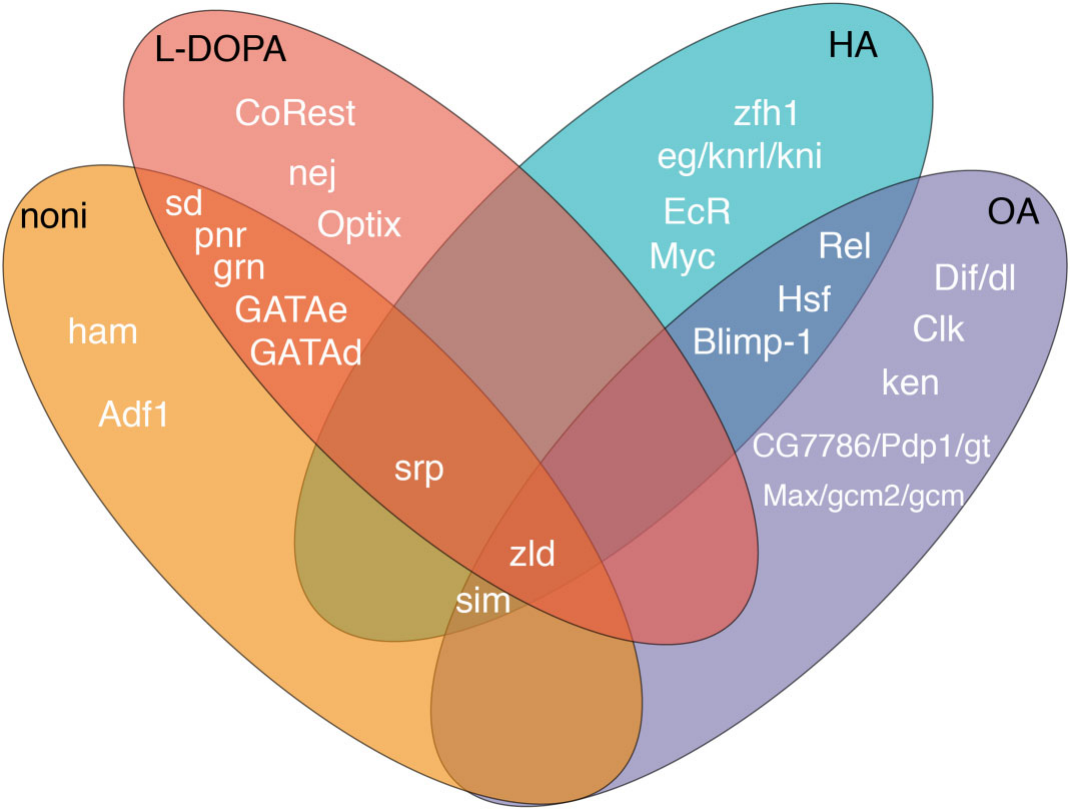
Several TFs are predicted to be involved in the response to multiple components of noni fruit. TFs predicted by i-*cis*Target analysis to regulate DEG expression in noni, l-DOPA, HA, and OA treatments in *D. sechellia* are shown. The GATA family of TFs (pnr, grn, GATAe, GATAd, and srp) is predicted to regulate DEG expression in both noni and l-DOPA treatment, with srp also being predicted in HA treatment. Zelda is predicted to regulate expression of DEGs in all 4 treatments. Single-minded is predicted to regulate DEG expression in noni, l-DOPA, and OA treatment. Rel, Hsf, and Blimp-1 are predicted to regulate DEG expression in both OA and HA treatments. Predicted regulatory networks for each treatment are found in Supplementary Fig. 3.

Significantly DEGs identified in previous studies that examined OA, l-DOPA, and HA exposure in adult female *D. sechellia* flies (Lanno *et al.* 2017, 2019; Drum *et al.* 2022) and were used for i-*cis*Target analysis in addition to DEGs we identified here in response to noni and we predicted TFs that control the plasticity of these DEGs. For this analysis, all DEGs, both up- and downregulated were used for all 4 treatments (OA, l-DOPA, HA, and noni). The TF *zelda* (*zld*) was predicted to regulate the expression of genes in all 4 treatments (Fig. 5). All 5 GATA family of TFs (*grn*, *pnr*, *GATAd*, *GATAe*, and *srp*) were predicted to regulate expression in both noni and l-DOPA treatments, and *srp* was also predicted to regulate expression in HA treatment. *sim* (*single-minded*) was predicted to regulate expression in noni, OA, and HA treatments. The TFs *Relish* (*Rel*), *Hsf*, and *Blimp-1* were predicted to regulate expression in both OA and HA treatments. Additionally, *GATAd* expression was significantly increased in l-DOPA treatment, *sim* expression was significantly increased in both l-DOPA and noni treatments, and *dl* expression was significantly increased in l-DOPA treatment (Supplementary Tables 1 and 2). Predicted regulatory networks for each treatment can be found in Supplementary Fig. 3.

## Discussion

Examining an organism’s genome-wide gene expression response to different environmental conditions can inform how organism physiology is modified to increase fitness in an environment-specific manner (Coolon *et al.* 2009; De Nadal *et al.* 2011). Comparing the physiology that allows *Drosophila sechellia* to feed on toxic *M. citrifolia* fruit to generalist susceptible sister species is an excellent model to study how insects evolve to specialize on toxic resources (Vogel *et al.* 2014). The chemicals found in noni fruit have driven the specialization of *D. sechellia* to its host, as *D. sechellia* has become resistant to the toxic volatile OA, is attracted to the fruit by toxic volatile HA, and utilizes consumed l-DOPA found in noni fruit to facilitate dopamine biosynthesis (Lavista-Llanos *et al.* 2014). Previous studies examined the whole organism transcriptional response to these separate components of noni fruit that has driven specialization (Lanno *et al.* 2017, 2019; Drum *et al.* 2022), and by comparing these transcriptional responses to the transcriptional response to noni fruit alone, we can better understand which responses are specific and which are more general in the evolution of *D. sechellia* noni specialization.

Genes involved in reproductive processes are upregulated in response to noni, l-DOPA, and HA, and genes involved in egg chorion formation are significantly enriched in noni treatment, but significantly downregulated in OA treatment. To better understand how *D. sechellia* has specialized on ripe noni fruit, which contains OA and HA volatiles, understanding the transcriptional response to all components in noni fruit together is necessary. *Drosophila sechellia* selectively oviposits on noni fruit and this increase in proteins involved in egg formation and development is consistent with the evolved reproductive traits for this species when it feeds on noni fruit (R’Kha *et al.* 1997; Lavista-Llanos *et al.* 2014).

The expression of many rRNAs is significantly downregulated in response to both OA and noni treatment, whereas several rRNAs are upregulated in response to HA treatment (Lanno *et al.* 2017; Drum *et al.* 2022). rRNA synthesis is induced by Ras/Erk signaling in *Drosophila* (Sriskanthadevan-Pirahas *et al.* 2018), and Myc and Max are predicted to regulate DEG expression in HA and OA treatments, respectively (Fig. 5). Future work examining how rRNA synthesis and protein translation are involved in the specialization of *D. sechellia* to noni fruit may elucidate a role for these genes in specialization on noni.

Previous studies examining gene expression responses in *D. sechellia* to the volatile fatty acids in noni fruit have focused on examining all of the DEGs in response to either OA or HA treatment to identify genes involved in evolved toxin resistance (Lanno *et al.* 2017, 2019; Drum *et al.* 2022). Interestingly, only 19.7% and 28.9% of DEGs found in these studies are responding specifically to OA or HA treatment, respectively, and only 2 genes are found that respond to both OA and HA treatment but no other treatments. In order to better understand how insects evolve gene expression responses to plant secondary defenses, it is helpful in laboratory studies to not only examine the response to the toxic chemical but to examine the wider context of response that more accurately portrays how this interaction would happen in nature where all compounds are experienced simultaneously, similar to that previously described in other species (Govind *et al.* 2010; Bansal *et al.* 2014; Hoang *et al.* 2015; Crava *et al.* 2016; De Panis *et al.* 2016; Birnbaum *et al.* 2017; Schweizer *et al.* 2017). The genetic basis of the resistance of *D. sechellia* to OA is polygenic, with the locus conferring the most resistance to OA residing on chromosome 3R (Hungate *et al.* 2013). Previous work has shown that the knockdown of *Osi6*, *Osi7*, and *Osi8* in adulthood, which reside on this locus, drastically decreases survival to OA (Andrade Lopez *et al.* 2017). Expression of *Osi6* and several other *Osiris* genes are significantly increased in *D. sechellia* in response to OA (Lanno *et al.* 2017), and *Osi6* is one of the 25 DEGs found only in OA and not in response to any of the other noni components. However, *esterase 6* (*Est-6*) RNAi has been previously shown to alter survival in *D. melanogaster* exposed to OA and the activity of esterase enzymes has previously been shown to be involved in OA resistance (Lanno *et al.* 2017, 2019; Lanno and Coolon 2019). Interestingly, *Est-6* expression is significantly enriched in response to l-DOPA and noni treatments, but not upon OA or HA exposure. This result may suggest that gene expression responses to the nontoxic chemicals found in the plant may be utilized as an indicator of toxin presence and therefore eliciting a gene expression response to help overcome the toxic chemicals that would also be found in the plant. As these genes expression response to each of these chemicals evolved together in response to exposure to all components simultaneously, it may be that responses to one chemical confer trait differences important for life in the presence of different components found in noni fruit.

Most TFs in our data set are expressed lowly and change little but small changes in TF abundance can alter gene expression in response to external stressor or chemical and may be observable by looking at changes in expression of downstream targets of TFs by RNA-seq. In order to predict which TFs are regulating the transcriptional response to noni in *D. sechellia*, we utilized i-*cis*Target to analyze DEGs to find shared TF binding motifs between DEGs. Expression of each predicted TF was analyzed in response to each treatment, and only a handful are significantly differently expressed between control and treatment measured by RNA-seq (Supplementary Tables 1–4).

All 5 members of the GATA family of TFs are predicted to regulate the expression of DEGs in noni and l-DOPA treatment, along with *srp* being predicted to also regulate DEG expression in HA treatment, but only *GATAd* is significantly upregulated in l-DOPA treatment. U-shaped, the Friend of GATA protein that binds GATA family of TFs that has been previously shown to be involved in fatty acid metabolism (Lenz *et al.* 2021) is significantly upregulated in l-DOPA treatment. Expression of GATA factors may be responding to noni and l-DOPA treatment in order to help metabolize volatile fatty acids found in noni fruit that are concurrently experienced by *D. sechellia* in their natural environment when feeding on noni fruit.

*Dorsal* (dl), a TF involved in Toll immune signaling in *Drosophila* (Valanne *et al.* 2011), is significantly upregulated only in l-DOPA treatment (Supplementary Table 2). Interestingly, no immune processes are significantly enriched upon l-DOPA exposure in *D. sechellia*, but are significantly downregulated upon OA and HA exposure. Of the genes significantly differentially expressed in all 4 treatments, many are involved in immune processes. As genes involved in insect immunity are being downregulated in response to OA and HA, future studies examining how the insect microbiome may play a role in the specialization of *D. sechellia* to noni fruit and perhaps resistance to its volatile fatty acids are needed. Gene regulatory response to fatty acids in concert with other components of noni fruit may be involved in regulating the immune system to facilitate the interaction of *D. sechellia* with its toxic host. *sim*, a TF that has been previously shown to be a repressor involved in nervous system development (Thomas *et al.* 1988; Estes *et al.* 2001), is significantly upregulated in both noni and l-DOPA treatments (Supplementary Fig. 1). In our network prediction, *sim* is predicted to regulate genes responding to OA, HA, and noni treatments, making it an excellent candidate for a master regulator that evolved to facilitate *D. sechellia* host specialization.

Examining the TFs that are predicted to regulate *Osiris* gene expression may help elucidate how they are regulated in response to OA in *D. sechellia*. From our network analysis of genes responding to OA exposure in *D. sechellia*, the TFs *Ken and Barbie* (*Ken*) and *Blimp-1* are predicted to regulate the expression of *Osi6*, one of the *Osiris* genes that was upregulated upon OA exposure in *D. sechellia* and shown to be involved in resistance to OA toxicity (Andrade Lopez *et al.* 2017; Lanno *et al.* 2017). More closely examining possible interaction(s) between these TFs and *Osiris* genes may shed light on their role in OA resistance.

Separating out genes involved in fruit metabolism compared to genes involved in responding to toxic substances is important for understanding this interaction. Previous work has shown that specialist fruit flies *D. sechellia* and *D. elegans* live significantly longer on protein-rich foods than generalist sister species (Watada *et al.* 2020). Noni fruit has a low amount of sugar and is relatively nutrient poor compared to other fruits (Singh *et al.* 2012), so understanding how *D. sechellia* has specialized to use this resource for feeding and breeding and if their transcriptional response plays a role in the metabolism of noni may shed light on how animals alter metabolism to specialize on nutrient poor sources. Examining the potential role of predicted TFs and other DEGs may help us understand the transcriptional response of *D. sechellia* to noni fruit and shed light on the genetic basis of *D. sechellia* evolved specialization on its toxic host plant. Using network prediction tools to understand the regulatory environment of gene expression may elucidate how gene regulation is being altered that would be missed in the analysis of DEGs alone, especially when there are hundreds of DEGs to analyze. Using a combination of transcriptome sequencing with methods to predict which TFs are responsible for gene expression responses due to external compounds may elucidate how insects are able to adapt to harsh environments and evolve to specialize on new and frequently toxic host plant species.

## Supplementary Material

jkac153_Supplemental_Tables

jkac153_Supplemental_Figure

## Data Availability

All RNA-seq data generated in this article have been submitted to the NCBI Gene Expression Omnibus under accession number GSE205467. Supplemental material is available at *G3* online.
