# Supplementary material for: Genomics analysis of Drosophila sechellia response to Morinda citrifolia fruit diet
Source: G3 (Bethesda). 2022 Jun 23;12(10):jkac153. doi: 10.1093/g3journal/jkac153 (PMC9526069; doi:10.1093/g3journal/jkac153)
Supplement: jkac153_Supplemental_Figure [file jkac153_supplemental_figure.zip › jkac153_Supplemental_Figure.docx]

**Supplemental Figure Legends:**

**Figure S1, Change in expression of predicted TFs in response to noni treatment.** A. Transcription factors predicted by i-*cis*Target to regulate the expression of upregulated DEGs in noni treatment and the mean of the log_2_ Fold Change is shown. None of these transcription factors are significantly differentially expressed in response to noni treatment. B. Transcription factors predicted by i-*cis*Target to regulate the expression of downregulated DEGs in noni treatment and the mean of the log_2_ Fold Change is shown. *Sim* is the only of these transcription factors found to be significantly differentially expressed in response to noni treatment.

**Figure S2, Predicted gene regulatory networks (GRNs) from analysis of significantly differentially expressed genes (DEGs) using i-*cis*Target and visualized using Cytoscape**. A. Upregulated DEGs were analyzed and a predicted GRN was assembled, predicting the transcription factors (TFs) *Adf1, EcR, gcm, Hey, Mad, RPII215, Sidpn, sima,* and *sr* (red) to be regulating the expression of upregulated DEGs (purple). Other known targets of these TFs that are not significantly differentially expressed are shown (green). B. Downregulated DEGs were analyzed and a predicted GRN was assembled, predicting the transcription factors *GATAd, GATAe, grn, ham, pnr, sd, sim, srp, zfh1,* and *zld* (red) to be regulating the expression of downregulated DEGs (green). Other known targets of these TFs that are not significantly differentially expressed are shown (orange).

**Figure S3. Predicted gene regulatory networks for each treatment are shown.** Predicted transcription factors are shown in green, upregulated DEGs are shown in red, downregulated DEGs are shown in blue, and other targets of these TFs are shown in yellow.
