# Supplementary figures and images for: Genomics analysis of Drosophila sechellia response to Morinda citrifolia fruit diet
Source: G3 (Bethesda). 2022 Jun 23;12(10):jkac153. doi: 10.1093/g3journal/jkac153 (PMC9526069; doi:10.1093/g3journal/jkac153)

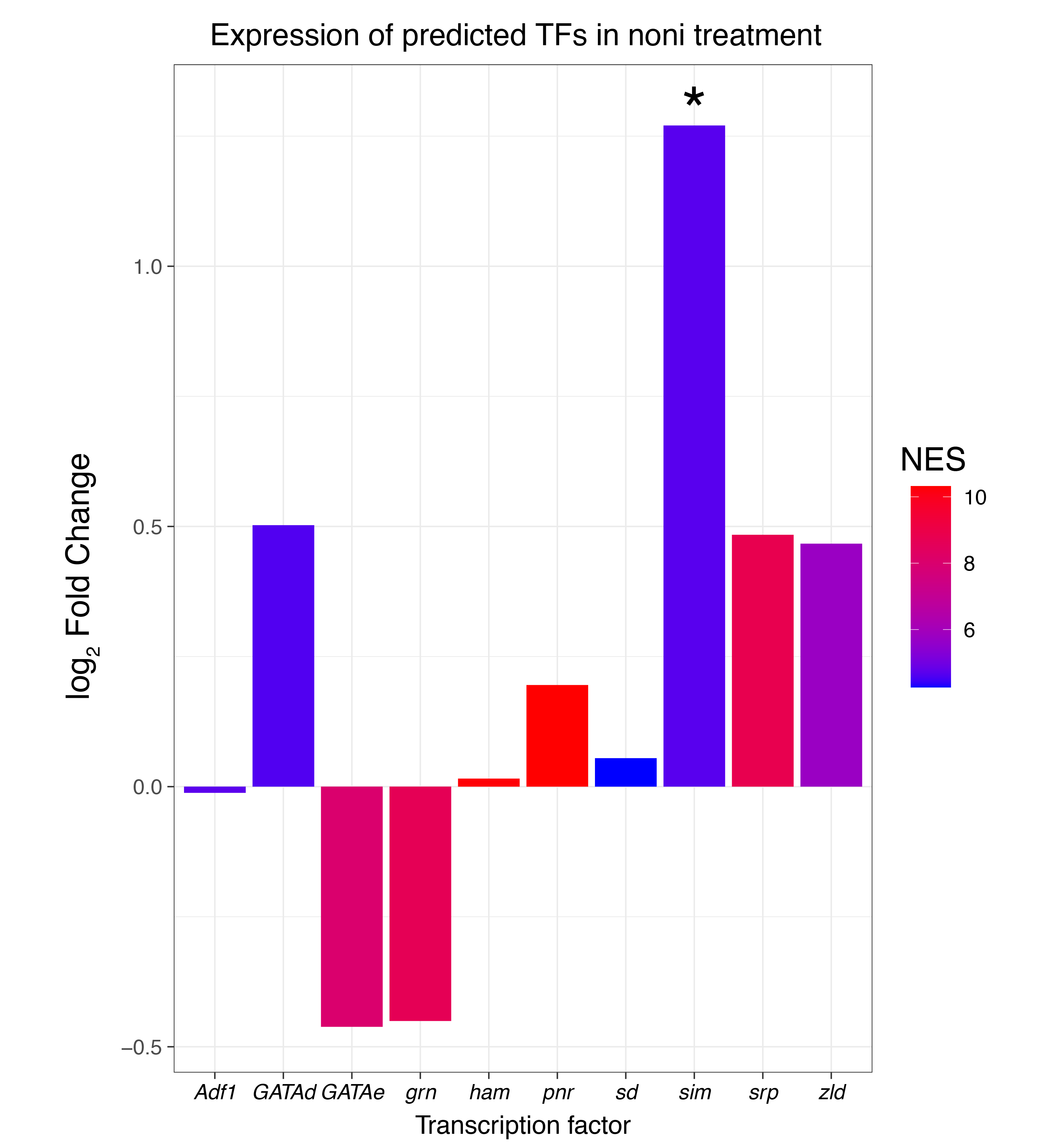

Supplement: jkac153_Supplemental_Figure [file jkac153_supplemental_figure.zip › Supplementary Fig. 1.png]
